# Nutritional Support for Alcoholic Liver Disease

**DOI:** 10.3390/nu15061360

**Published:** 2023-03-10

**Authors:** Tomoko Tadokoro, Asahiro Morishita, Takashi Himoto, Tsutomu Masaki

**Affiliations:** 1 Department of Gastroenterology and Neurology, Faculty of Medicine, Kagawa University, Kita 761-0793, Kagawa, Japan; 2 Department of Medical Technology, Kagawa Prefectural University of Health Sciences, Takamatsu 761-0123, Kagawa, Japan

**Keywords:** alcoholic liver disease, alcoholic cirrhosis, protein–energy malnutrition

## Abstract

Malnutrition is a common finding in alcohol use disorders and is associated with the prognosis of patients with alcoholic liver disease (ALD). These patients also frequently show deficiencies in vitamins and trace elements, increasing the likelihood of anemia and altered cognitive status. The etiology of malnutrition in ALD patients is multifactorial and complex and includes inadequate dietary intake, abnormal absorption and digestion, increased skeletal and visceral protein catabolism, and abnormal interactions between ethanol and lipid metabolism. Most nutritional measures derive from general chronic liver disease recommendations. Recently, many patients with ALD have been diagnosed with metabolic syndrome, which requires individualized treatment via nutritional therapy to avoid overnutrition. As ALD progresses to cirrhosis, it is frequently complicated by protein–energy malnutrition and sarcopenia. Nutritional therapy is also important in the management of ascites and hepatic encephalopathy as liver failure progresses. The purpose of the review is to summarize important nutritional therapies for the treatment of ALD.

## 1. Introduction

**Figure 1:**
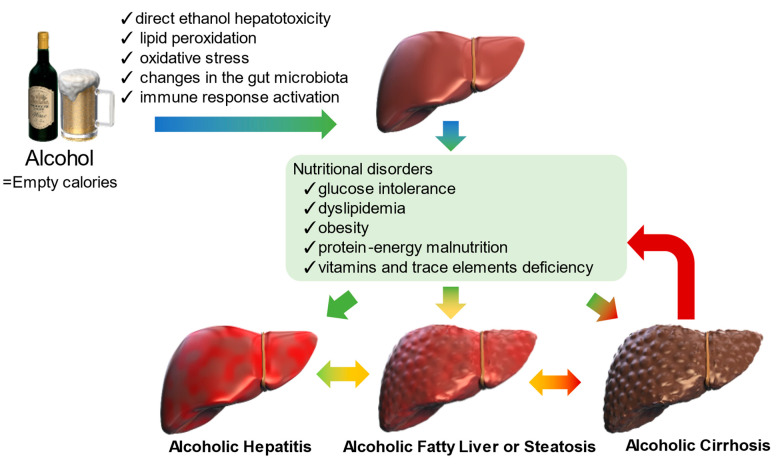
Nutritional disorders and liver disease in heavy alcohol drinkers.

Alcohol use disorders (AUDs) are among the leading causes of preventable diseases and liver disease-related deaths in the United States and the world. However, the prevalence of AUD and alcohol-related liver disease (ALD) has markedly increased over the past few years [1]. ALD is caused by drinking an average of ≥60 g/day of pure alcohol for at least 5 years. The onset and progression of ALD is influenced by many factors, including age, sex, presence of an underlying disease, genetic predisposition, immune function, obesity, and overnutrition. Complete abstinence is the cornerstone of treatment, improving clinical outcomes in all ALD stages [2]. Malnutrition is present in approximately 50% of outpatients with ALD and nearly all inpatients with ALD; importantly, it adversely affects treatment responses and patient outcomes [2,3]. The degree of nutritional impairment varies according to the stage of ALD progression, and nutritional therapy should be individualized to the disease type. As shown in Figure 1, ALD is histologically classified into three stages [4]: (i) alcoholic fatty liver or steatosis wherein fat accumulates in the liver parenchyma; (ii) alcoholic hepatitis (AH) wherein liver cells are inflamed and the clinical outcomes depend on the severity of the damage; and (iii) alcoholic cirrhosis wherein liver damage is irreversible, leading to complications of cirrhosis and portal hypertension.

There are various mechanisms of alcohol-induced hepatocellular damage [5]. The ingested alcohol is oxidized by alcohol dehydrogenase (ADH) and cytochrome P450 2E1 (CYP2E1) to acetaldehyde, which is converted to acetic acid by aldehyde dehydrogenase (ALDH). Acetaldehyde causes hepatocellular damage. Reactive oxygen species (ROS) generated during alcohol metabolism inhibit antioxidant activity in hepatocytes [6]. Markers of oxidative stress include serum NADPH oxidase (NOX2) [7] and urinary 8-isoPGF2α [8]. Alcohol-induced changes in the gut microbiota may contribute to alcohol-induced oxidative stress and intestinal hyperpermeability [9]. Microbial therapy such as probiotics improve ALD by restoring normal intestinal flora and strengthening the intestinal barrier [10]. Activation of Kupffer cells by intestine-derived lipopolysaccharides is also a major factor in ALD. Lipopolysaccharide is recognized by the Toll-like receptor (TLR) 4 complex, which is expressed on immune cells as well as parenchymal cells, and induces activation of inflammatory cytokines. Chronic alcohol exposure sensitizes Kupffer cells to lipopolysaccharide-induced inflammatory cytokine production [5,11]. Additionally, alcohol can cause hepatotoxicity directly or indirectly through its metabolites by various other mechanisms. Edible plants such as fruits, vegetables, spices, grains, and teas have been reported to be effective against ALD via these various mechanisms [12].

ALD refers to a series of diseases that begin with fatty liver and progress to AH and alcoholic cirrhosis. Malnutrition in patients with ALD is correlated with decreased quality of life, increased risk of infection, frequent hospitalization, complications, mortality, and financial burden. Physicians, including gastroenterologists and hepatologists, should be familiar with the assessment and management of malnutrition and nutritional supplementation [13]. Alcoholic beverages contain little or no protein, vitamins, trace elements, fiber, or other nutrients [14]. However, resting energy expenditure (REE) is usually elevated in AUD patients, even though they often do not eat and only drink alcohol. Malnutrition due to impaired digestion and nutrient absorption from alcohol and organ damage are common complications in patients with conventional ALD. These patients are predominantly characterized by decreased body weight, muscle or fat mass, muscle strength, visceral protein levels, and immune function [15]. Thus, patients with ALD often develop protein–energy malnutrition (PEM). The occurrence of malnourishment in ALD patients is a multifactorial event that includes altered olfactory and taste perception, appetite-related hormonal changes, and altered gut flora [2]. Patients admitted with severe AH often have a history of heavy alcohol consumption. Thus, management during hospitalization should focus on nutritional support in addition to alcohol withdrawal, treatment of infection and sepsis and complications of cirrhosis and portal hypertension, and specific treatment of AH [16,17]. 

Meanwhile, with the recent increase in the number of ALD patients with obesity, it has been noted that overnutrition conditions, such as excessive alcohol consumption, obesity, and hyperglycemia are closely related to the pathological progression of ALD. Alcohol consumption can lead to obesity, especially when accompanied by excessive fatty food intake and lack of exercise [14]. In recent years, patients with ALD have also shown a high frequency of obesity and overnutrition, and lifestyle-related diseases are known to promote disease progression. Therefore, it is required to take measures to prevent lifestyle-related diseases. In an observational study of Japanese male patients with ALD, obesity was found in 26%, diabetes in 34%, hypertension in 23%, and dyslipidemia in 23% of patients [18]. A U-shaped curve was also reported, in which the risk for type 2 diabetes was lower for small-volume drinkers than for nondrinkers, and the risk increased with increasing alcohol consumption [19]. Evidence for the health benefits of small amounts of alcohol consumption has remained controversial in recent years. All-cause mortality increased with increasing alcohol consumption, and only zero drinks minimized harm to overall health outcomes [20]. Thus, there is no basis for recommending small amounts of alcohol consumption to extend life expectancy, and the most important management of ALD is abstinence from alcohol.

However, advanced ALD is often irreversible and does not improve with alcohol abstinence alone. In the management of patients with ALD, it is important to assess and provide guidance on lifestyle habits such as diet, along with reducing alcohol consumption. As shown in Table 1, the recommended energy and protein levels vary by ALD stage (Table 1). Patients with ALD should receive appropriate nutritional assessment and nutritional support [21]. The Nutrition Risk Screening 2002 and Malnutrition Universal Screening Tool are recommended tools to assess nutritional status. They are valid tools for screening hospitalized patients for the risk of malnutrition and are recommended by the European Society for Clinical Nutrition and Metabolism [22,23]. The Royal Free Hospital-Nutritional Prioritizing Tool is also considered excellent for predicting malnutrition risk in cirrhosis patients [24]. In this article, we review the nutritional therapies that health care providers should be aware of when treating patients with ALD.

## 2. Methods

In this review, to identify all relevant studies, the authors searched electronic databases for currently published studies. The published articles were retrieved via PubMed and MDPI from available peer-reviewed journals. The search was conducted using keywords related to ALD and nutritional therapy, such as alcohol, alcoholic hepatitis, steatosis fatty liver, cirrhosis, or nutrient (n = 10,798). Following the initial search, the articles’ reference lists were reviewed, and potentially eligible articles were selected. We have selected literature that can be viewed in full text. Articles that were not reported in English or those in which the participant was diagnosed with a liver disease other than ALD were excluded. Quality assessment and data extraction were performed by two reviewers independently. Finally, 100 papers were extracted.

Ethanol is empty calories, and prolonged alcohol consumption without adequate dietary intake produces a variety of nutritional disorders. Possible mechanisms of alcohol-induced liver injury include direct ethanol hepatotoxicity, lipid peroxidation, oxidative stress, changes in the intestinal microbiota, and immune response activation. These nutritional disorders can affect any stage of alcoholic hepatitis, alcoholic fatty liver or steatosis, and alcoholic cirrhosis. In addition, the pathogenesis of liver damage, especially cirrhosis, further exacerbates nutritional disorders.

## 3. Alcohol-Induced Metabolic Abnormalities

### 3.1. Glucose Intolerance

Metabolic syndromes, including diabetes, have a significant involvement in the development of liver fibrosis and hepatocellular carcinoma [29,30]. Alcohol causes increased gluconeogenesis in the liver, increased glycogenolysis, and inhibition of insulin secretion from the pancreas, all of which contribute to diabetes mellitus [31]. Accordingly, type 2 diabetes mellitus is a common complication in ALD patients. The effect of moderate alcohol consumption on liver enzymes increases with increasing body mass index (BMI) [32]. The incidence of glucose intolerance associated with alcoholic fatty liver disease has increased in recent years [33]. In men with a BMI of <22 kg/m^2^, moderate (23.0 g/day) or high alcohol intake has been reported to predominantly increase the risk of developing diabetes. Patients at risk for alcohol consumption may have poor adherence to diabetes treatment, leading to higher morbidity and mortality in these patients. Adverse events during diabetes treatment can also be induced by alcohol consumption. The potential of alcohol to induce hypoglycemia is greater in the presence of sulfonylureas. Alcohol consumption also increases the risk of metformin-induced lactic acidosis.

### 3.2. Dyslipidemia

Approximately 30% of alcohol consumed is absorbed into the body through the stomach while the remaining 70% is absorbed through the jejunum, and more than 90% is transported to the liver via the portal vein. Alcohol is then metabolized by the alcohol dehydrogenase (ADH)-mediated system; microsomal ethanol oxidizing system, which is a non-ADH system; and nicotinamide-adenine dinucleotide phosphate oxidase–catalase reaction system, which is a non-ADH system [34]. Prolonged excessive drinking induces the secretion of enzymes that increase alcohol and aldehyde metabolism. Recent studies have shown that alcoholic fatty liver is partially CYP2E1 dependent [35]. The release of free fatty acids (FFA) from fatty tissues other than the liver into the bloodstream is increased, and excess FFA are converted to triglycerides (TG) in the liver where it accumulates. Fatty liver is exacerbated by alcohol metabolism, in which FFA β-oxidation in the mitochondria is suppressed. In alcoholic fatty liver, the expression of microsomal TG transfer protein, which releases TG from the liver into the blood, is decreased [36], and lipid synthesis and consumption in hepatocytes are unbalanced, further contributing to intrahepatic fat accumulation. There are reports that miRNAs are involved in ALD-induced hepatic fat accumulation [37,38], and further studies are warranted.

### 3.3. Obesity

Among patients with ALD, 44.5% and 32.4% are obese and have metabolic syndrome, respectively. These conditions have also been shown to be independent prognostic factors in patients with chronic liver disease, including ALD [39]. Further, obesity and alcohol consumption synergistically contribute to liver carcinogenesis [40,41]. Obesity persisting for >10 years has been reported to be an independent risk factor for fatty liver, acute AH, and cirrhosis [42]. Excessive alcohol consumption is associated with adipose tissue inflammation and fatty liver, and adipose tissue secretion and adipokines may be involved in the development of ALD. Elevated adiponectin was associated with advanced liver dysfunction in patients with ALD [43], and it has been suggested that visceral fat accumulation may be involved in the progression of ALD. Obesity, liver iron excess, and diabetes are also independent risk factors for fibrosis in ALD and have high therapeutic significance in clinical management [44]. A new concept of fatty liver disease named metabolic dysfunction-associated fatty liver disease (MAFLD) has been recently proposed to describe fatty liver complicated by metabolic dysfunction, regardless of the amount of alcohol consumption [45,46]. MAFLD studies have shown that alcohol consumption and metabolic abnormalities are independent factors associated with liver fibrosis and cardiovascular events, respectively [47]. In MAFLD patients, even mild alcohol consumption is associated with worsening of liver fibrosis [48]. However, there is currently no global consensus on MAFLD, and this is one of the issues to be considered.

### 3.4. Protein–energy Malnutrition

The liver is a major organ for nutrient metabolism, and PEM is commonly observed in chronic liver disease and correlates with ALD progression and liver dysfunction. Sarcopenia in alcoholic liver disease has two primary causes: liver disease and direct effects of ethanol [15]. In chronic liver disease, glycogen stores in the liver are depleted, and sugar is not available as an energy source, resulting in the breakdown of protein and lipids from muscle and adipose tissue and worsening nutritional status. In addition, the essential branched-chain amino acids (BCAAs) are involved in albumin synthesis and the maintenance of skeletal muscle mass, and thus, a decrease in BCAAs is also closely related to the development of sarcopenia in chronic liver disease [49,50]. Protein metabolism is assessed using serum albumin levels, the BCAA-to-tyrosine ratio (BTR), and albumin–bilirubin score [51], and their abnormal values are considered an indication for nutritional intervention for PEM. As PEM progresses, nutritional impairment is more frequent and is closely related to prognosis [52]. Previously, the importance of a rested, high-protein, high-energy diet for cirrhosis was emphasized. However, nutritional interventions for both muscle loss and obesity, represented by “sarcopenia obesity,” have become an important issue in recent clinical practice for ALD because even obese patients can be undernourished.

## 4. Nutritional Therapy for ALD

### 4.1. Total Calories, Carbohydrates, Fats, and Proteins Recommended for ALD

#### 4.1.1. Total Calories and Nutritional Support Route Recommended for ALD

Total caloric intake should be at least 1.2 to 1.4 times the resting energy expenditure, generally 30 kcal/kg or more [53]. Resting energy expenditure is usually increased in cases of AH and cirrhosis. In the presence of glucose intolerance or obesity, caloric requirements may be lower and require individualized attention. The first choice for nutrition is spontaneous oral intake. If oral intake is difficult, enteral nutrition should be preferred. Intravenous nutrition is considered the last choice because of the possibility of an infection.

#### 4.1.2. Carbohydrates Recommended for ALD

Carbohydrates are recommended to constitute approximately 50–60% of the total calories (preferably as complex carbohydrates) [54]. However, recommendations vary depending on the pathophysiology of ALD and the presence or absence of glucose intolerance [54]. Among carbohydrates, excessive intake of fructose, which is commonly used commercially in soft drinks and other products, should be avoided [55]. Fructose in food and drink has been significantly correlated with the degree of inflammation and fibrosis, and is also a risk factor for the development of liver cancer [56].

#### 4.1.3. Fats Recommended for ALD

Dietary fat has been implicated in the pathogenesis of ALD. Fat should comprise 30–35% of the total calories, be high in unsaturated fatty acids, and contain sufficient amounts of essential fatty acids [54]. For instance, MCT oil has recently attracted attention as it has been reported to improve liver function in an NAFLD model [57]. Increasingly, reports indicate that n-3 polyunsaturated fatty acids are useful in alleviating ALD through multiple mechanisms [58]. Fish oil is rich in n-3 polyunsaturated fatty acids such as eicosapentaenoic acid (EPA) and docosahexaenoic acid (DHA) and shows protective effects against fatty liver by lowering blood triglyceride levels in hypertriglyceridemic patients [59]. Fish oil substitution increased plasma adiponectin concentrations and promoted fatty acid oxidation in the liver. In addition, the addition of fish oil promoted hepatic autophagy and lipid degradation, and subsequently inhibited the accumulation of lipids in the liver. It also reduced lipid peroxidation [58]. The addition of unsaturated fatty acids protects various mitochondrial enzymes by reducing oxidative stress and prevents alcohol-induced fatty liver and mitochondrial dysfunction in animal models [60].

#### 4.1.4. Proteins Recommended for ALD

The recommended level of daily protein intake is approximately 1.0–1.5 g/kg/day, depending on the pathology [54]. Patients with stable liver disease were able to eat a standard protein-containing diet and did not experience increased hepatic encephalopathy [61]. In case of cirrhosis, BCAAs should be included. The following sections describe specific nutritional therapies for each stage of ALD.

#### 4.1.5. Alcoholic Hepatitis

AH is an acute form of hepatitis associated with serious morbidity and mortality and may occur in patients with steatosis or cirrhosis. ALD has a multifactorial etiology including genetic factors, hepatocellular damage due to alcohol, reactive oxygen species, and microbial components of intestinal origin cause lipidification in the liver and mobilization and activation of inflammatory cells (macrophages and neutrophil leukocytes). Continued alcohol consumption and high levels of inflammatory cytokines induce the activation of stellate cells, leading to progressive fibrosis. The cardinal sign of AH is progressive jaundice, which is often associated with high fever, fatigue, weight loss, and malnutrition [62]. In an analysis of data from the American Veterans Study, undernourished patients with severe AH had higher morbidity and mortality rates. A clear association was found between low intake of regular food and high mortality [63]. Patients with a daily caloric intake of less than 21.5 kcal/kg/day had higher infection and mortality rates at 6 months than did those with higher intakes. Several studies have also reported that PEM is present in almost all patients with severe AH and is associated with a poor prognosis [63].

Nutritional therapy should be administered to all patients with severe AH who are unable to meet their nutritional requirements through voluntary food intake to improve survival, infection rates, liver function, and healing of encephalopathy [64]. Proper nutritional therapy improves the immunological status and has a beneficial effect against infections, which are often the cause of death in AH [65]. Among patients with AH, a high-protein, high-energy diet is important for those without gastrointestinal bleeding complications. Patients with severe AH should be managed with aggressive supportive care, and a high energy intake is associated with improved survival [5]. Furthermore, nutritional therapy has been shown to improve hepatic encephalopathy and reduce the risk of developing infections [66]. Severe AH patients are recommended to consume 1.2–1.5 g/kg protein and 35–40 Kcal/kg calories per day [25]. However, these goals are often difficult to achieve in the clinical setting [67]. The enteral route is the preferred route for nutritional support because of its low cost, high safety, and low risk of infection. However, data on the survival benefits of enteral or parenteral nutritional supplementation are controversial. 

Enteral nutrition can overcome the effects of anorexia and dysphagia but not enteral malabsorption. In addition, although it has also been shown to be beneficial, well-tolerated, and may improve liver function, its effects on the skeletal muscle and other nutritional parameters are inconclusive [68]. A recent randomized study showed no survival benefit from enteral nutritional supplementation in severe AH [21]. A randomized study of patients with severe AH treated with corticosteroids also found that enteral nutrition administered via feeding tubes for 14 days did not prolong survival because it was difficult to implement and had adverse events [69]. The study demonstrated that regardless of the assigned therapy, patients with a daily caloric intake of less than 21.5 kcal/kg had a significantly higher risk of mortality and infection at 1 and 6 months. Although parenteral nutrition might avoid the complications of nasogastric feeding, there is insufficient evidence to support a recommendation, particularly given that parenteral feeding is associated with a higher risk of line sepsis [67].

#### 4.1.6. Alcoholic Fatty Liver or Steatosis 

As with nonalcoholic steatohepatitis (NASH), many alcoholic fatty liver (AFL) cases are complicated by obesity associated with overnutrition [70]. The amount of fat deposits in the liver tissue is generally greater in NASH than in AFL. Initially, small droplet fatty deposition occur around the central veins and then spread to the periportal area as the disease progresses [71]. AFL occurs in 90% of heavy drinkers and resolves relatively easily with abstinence from alcohol consumption. Given that excessive alcohol consumption leads to fat accumulation in hepatocytes by suppressing gluconeogenesis, inhibiting fatty acid beta-oxidation, promoting fatty acid and triglyceride synthesis, and mobilizing free fatty acids from peripheral adipose tissue, alcohol abstinence, and lifestyle modification are important in patients with AFL. The recommended diet for AFL is the same as that for NASH: 20–30 kcal/kg/day and 1.0–1.5 g/kg/day of protein, with 20–25% protein, 50–60% carbohydrate, and 15–20% total energy from lipids with low saturated fatty acid intake. However, rapid weight loss can lead to liver damage, and it also promotes fat accumulation in the liver; therefore, a weight loss of 1–2 kg/month is recommended [26]. Nutritional disorders due to PEM appear during the progression from alcoholic fatty liver to cirrhosis. Considering that muscle mass loss due to BCAA deficiency and potential hepatic encephalopathy complications is frequently observed, it is advisable to start nutritional support therapy at an early stage. The European Society for Clinical Nutrition and Metabolism guidelines indicate that oral intake is more effective than tube or intravenous nutrition in enhancing intestinal mucosal barrier function and maintaining intestinal flora in patients with AFL, and thus leading to a decreased risk of infection and mortality [64]. Therefore, nutritional therapy for patients with AFL should be based on the grade of liver injury.

#### 4.1.7. Alcoholic Cirrhosis

Malnutrition is generally associated with cirrhosis and its severity [25]. Patients with alcoholic cirrhosis fall into a catabolic state of starvation more rapidly than normal individuals. Alcoholic cirrhosis patients who fast overnight are similar to healthy people who are completely starved for 2–3 days [72]. In patients with cirrhosis, fasting for 10–12 h can easily reduce the supply of sugar to the liver and lead to starvation. In daily life, this corresponds to evening and early morning hours. Shortening the time between meals and distributing them throughout the day is beneficial for patients with ALD and those with cirrhosis [15]. In addition, because of nutritional impairment in advanced cases of alcoholic cirrhosis, there are fewer obese cases than fatty liver cases, even when total energy intake exceeds consumption. Further, patients are often below the standard body weight, even when BMI is considered. PEM is a particularly common complication in patients undergoing invasive treatment for hepatocellular carcinoma or esophageal varices.

The basic nutritional intervention for cirrhosis is the same regardless of the cause of cirrhosis. For PEM (serum albumin level ≤ 3.5 g/dL), Child–Pugh class B or C, or sarcopenia, nutritional intervention with divided meals (3 to 5 meals per day), LES, and BCAA-containing foods are provided. If there is no improvement in nutritional status or cirrhotic complications develop, enteral nutrition for liver failure or BCAA formulas should be started promptly [73]. LES may also be useful in improving the pathogenesis of alcoholic cirrhosis. LES with BCAA-enriched enteral nutrition for liver failure in patients with cirrhosis is expected to increase serum albumin levels and improve long-term abnormalities in energy metabolism, glucose tolerance, and quality of life. The requirements for LES are as follows: (1) approximately 200 kcal; (2) good balance of carbohydrates, fats, and proteins; and (3) BCAA-containing foods. High-protein evening meals take advantage of night-time anabolic opportunities and may prevent muscle loss [74]. 

Long-term oral intake of a BCAA mixture is superior to a standard diet for improving serum albumin levels and energy metabolism [27]. Plasma free amino acid imbalances lead to a decrease in BCAA, a decrease in Fischer’s ratio, or BTR due to an increase in aromatic amino acids and an increase in methionine levels. Aromatic amino acids and methionine are metabolized in the liver, and their blood levels are increased with worsening severity of cirrhosis. BCAAs are normally metabolized in peripheral tissues such as muscle and adipose tissue. However, in cirrhosis, they are used as an energy source and for ammonia metabolism, and thus, their blood levels are reduced. Even in conditions considered to be compensated cirrhosis (e.g., Child–Pugh grade A), BCAAs are deficient. In contrast, tyrosine concentrations increase with progression of cirrhosis, resulting in a decrease in BTR [75].

Oral BCAA formulations are available in two forms: enteral nutrition for liver failure (or component nutrition for liver failure) and oral granules. All enteral nutritional formulations for hepatic insufficiency are high in the BCAAs valine (Val), leucine (Leu), and isoleucine (Ile), and regular dosage (2–3 packets/day) provides 11–17 g/day of BCAAs. These formulations are indicated for the treatment of hepatic encephalopathy in patients with noncompensated liver cirrhosis. Given that they contain the three major nutrients of proteins, carbohydrates, and fat, as well as vitamins and minerals, they are administered not only to improve hepatic encephalopathy but also to improve nutritional status. Meanwhile, the oral BCAA granule formulation is composed of BCAAs only, with Val, Leu, and Ile in a 1:2:1.2 ratio [76]. It is indicated for the improvement of malnutrition with hypoalbuminemia in patients with noncompensated liver cirrhosis without hepatic encephalopathy. Patients must be able to consume sufficient food. In a randomized trial of patients with alcoholic cirrhosis, skeletal muscle signaling abnormalities and the rate of muscle protein synthesis were restored by a single dose of a leucine-enriched BCAA mixture [77]. BCAA granules improved serum albumin levels in approximately 8 weeks and improved symptoms such as edema of the extremities, general malaise, fatigue, and muscle cramps. These benefits improved the quality of life in cirrhosis, reduced the frequency of hepatic encephalopathy, and prolonged life expectancy [78]. Nutritional therapy, including LES, not only in the short-term but also in the long-term on an outpatient basis, is associated with improved nutritional status and prognosis in cirrhosis [79].

However, obesity and the accumulation of subcutaneous adipose tissue are poor prognostic factors in patients with alcoholic cirrhosis, and nutritional therapy should be tailored to individual conditions [80]. Alcoholic cirrhosis results in a combination of undernutrition and overnutrition, which requires individualized management, including monitoring of nutritional status and nutritional guidance. Considering that enteral nutrition for liver failure contains approximately 200 kcal, overfeeding should be avoided by subtracting the amount of enteral nutrition from the total energy. The addition of LES without intervention may exacerbate glucose intolerance [81]. Energy intake is based on 25–35 kcal/kg (standard body weight)/day in the absence of glucose intolerance. In the presence of glucose intolerance, 25 kcal/kg/day and protein requirements are based on 1.0–1.5 g/kg/day (including BCAA preparations) in the absence of protein intolerance. Meanwhile, in the presence of protein intolerance, 0.5–0.7 g/kg/day plus enteral nutrition with high BCAA content for hepatic failure is recommended [28].

Studies on enteral nutrition for cirrhosis have not shown any benefits [82]. Short-term enteral nutrition after the treatment of esophageal varices has also not been shown to be beneficial [83]. Placement of a percutaneous endoscopic gastrostomy tube is generally not indicated in cirrhosis but may be considered in the absence of ascites, although its indication should be carefully considered [84]. Early nutrition with regular solid food was recently found to be safe in conscious patients with successful ligation of esophageal varices. It resulted in better nutritional status and was associated with a lower incidence of infection in bleeders than in delayed nutrition [85]. 

### 4.2. Vitamins and Trace Elements Recommended for ALD

#### 4.2.1. Overview

The development of alcoholic fatty liver, AH, and cirrhosis is a result of longstanding nutritional deficiencies that require supplementation with appropriate amino acids, vitamins, minerals, and other nutrients [86]. As shown in Table 2, severe ALD micronutrient deficiencies are expected in patients, and thus, supplementation is needed. Vitamins, which are molecules found in small amounts in various foods, are essential for normal metabolism, and deficient vitamin levels in the body can lead to serious diseases. Alcoholics are more likely to be deficient in certain vitamins, especially vitamins B1 (thiamine), B2 (riboflavin), B6 (pyridoxine), C (ascorbic acid), and folic acid, even in the absence of liver disease [14]. The primary cause of vitamin D deficiency is dietary deficiency; however, other factors may be involved. Alcoholism may affect the absorption, storage, metabolism, and activation of these vitamins [87]. Given the frequency of deficiencies in B vitamins, zinc, and vitamin D, supplementation may be beneficial. Vitamins and trace elements should be taken at least in the recommended daily amounts.

Patients with severe liver failure have low levels of zinc, vitamin E, and vitamin A and high levels of vitamin B12 and ferritin [88]. In severe ASH, oral administration of multivitamin and zinc formulations is reasonable because deficiencies occur frequently, and empirical oral supplementation is less expensive than laboratory measurements to establish deficiencies before supplementing individual micronutrients. Given that intravenous nutrition is likely to have a short duration, the risk of adverse events from long-term vitamin and micronutrient supplementation is low, even without measuring serum concentrations. Certain vitamins, including vitamins A, D, and K, should be administered with thiamine, folic acid, and pyridoxine to correct deficiencies [64,89].

**Table 2:** Deficiency or surplus of vitamins, major minerals, and trace elements in ALD.

Nutrients	ALD	Signs/Symptoms in Deficiency/Excess	Supplementation	Reference(s)
Vitamin A/Retinol	↓	Night blindness	It may be considered in case of deficiency	[90]
		Immune system disorders		[90,91]
		Severe liver damage		[91]
Vitamin D	↓	Bone diseases (including rickets in children and osteomalacia in adults)	Can be considered	[92]
		Risk of respiratory illness		[93]
		Altered gut barrier/immune function		[94,95,96]
Vitamin E	→-↓	Related to oxidative stress	Can be considered	[97]
Vitamin B1/Thiamine	↓	Wernicke–Korsakoff syndrome	Can be considered	[98]
		Neurologic symptoms		[98]
		Cardiovascular abnormalities		[98]
Folate	↓	Anemia	Can be considered	[99]
		Altered methylation		[100]
		Increased risk of hepatocellular carcinoma		[101,102]
Zinc	↓	Immune abnormalities	It may be considered in case of deficiency	[103]
		Insulin resistance		[104]
		Hepatic encephalopathy		[105,106]
Magnesium	↓	Insulin resistance	Can be considered	[107]
		Hypocalcemia or hypokalemia		[108]
		Muscle cramps		[109]
Iron	↓-↑	Increased risk of hepatocellular carcinoma (surplus)	It may be considered in case of deficiency	[110,111]
		Liver fibrosis (surplus)		[112]
		Insulin resistance (surplus)		[113]
Manganese	↑	Hepatic encephalopathy	No evidence	[114,115]
Copper	↓-↑	Central nervous system dysfunction (deficiency)	No evidence	[68]
Selenium	↓	Ballooning of hepatocytes	Can be considered	[116]

↓ means deficiency, → means normal range, and ↑ means surplus.

#### 4.2.2. Vitamin A (Retinol)

Hepatic vitamin A levels are decreased in alcoholics [117] and vitamin A deficiency has been a longstanding problem [14]. Vitamin A deficiency is particularly common in patients with cirrhosis and is caused by both reduced dietary intake and decreased absorption of vitamins. Alcohol consumption has a significant impact on systemic retinoid homeostasis [117]. Vitamin A deficiency is associated with night blindness, immune system disorders, and severe liver diseases. Alcohol affects the level and metabolism of vitamin A and β-carotene in the body in several ways. For example, alcohol increases vitamin A levels in some tissues and decreases them in other tissues. In addition, alcohol hastens or alters the conversion of vitamin A into other compounds. Some or all of these changes may contribute to the toxic effects of alcohol on the liver and the development of liver fibrosis [118]. Serological vitamin A deficiency is often seen in patients with cirrhosis and is associated with liver injury. Patients with vitamin A deficiency have higher rates of ascites, hepatic encephalopathy, and hepatorenal syndrome during follow-up. Clinical complications and infections also occur more frequently in cirrhosis patients with vitamin A deficiency [119]. However, excessive vitamin A intake also has detrimental effects. Therefore, no clear conclusions regarding vitamin A supplementation have been reached. Prospective clinical trials on therapeutic vitamin A are required. The concern about hepatotoxicity due to overdose should be addressed.

#### 4.2.3. Vitamin D

Vitamin D deficiency has also been reported in alcoholics [120]. It causes bone diseases including rickets in children and osteomalacia in adults, respiratory illness, and small-for-gestational age births. In addition, it is related to an altered gut barrier and immune function [95]. Vitamin D deficiency has been suggested to exacerbate hepatic oxidative stress and inflammation during chronic alcoholic liver injury [121]. Hypovitaminosis D is an independent risk factor of sarcopenia in patients with chronic liver disease [122]. Vitamin D deficiency is prevalent in as many as 64–92% of patients with cirrhosis, and this rate increases with the severity of liver failure [123]. Low 25(OH)D levels have been associated with increased ALD mortality [124]. The European Association for the Study of the Liver guidelines do not have absolute recommendations for patients with chronic liver disease except for those with chronic cholestasis. However, it is reasonable to supplement oral vitamin D in all chronic liver disease patients with vitamin D levels below 20 ng/mL until serum vitamin D levels exceed 30 ng/mL [67]. Oral administration of native vitamin D3 at a dose of 2000 IU once daily for 12 months has been reported to improve sarcopenia in patients with noncompensated liver cirrhosis. Vitamin D administration may be an effective and safe treatment option to increase or restore skeletal muscle mass and strength or to prevent loss of muscle mass and strength [125]. Adverse reactions to excessive vitamin D intake include gastrointestinal problems, drowsiness, headache, muscle pain, thirst, weakness, and kidney stones, most of which are associated with hypercalcemia [126]. In contrast, vitamin D toxicity is rare unless serum 25(OH)D levels are greater than 150 ng/mL [127]. As such, vitamin D supplementation is likely to be beneficial.

#### 4.2.4. Vitamin E

Vitamin E contributes to the histological improvement of liver injury in NASH [128]. Alcoholics with cirrhosis tend to have low vitamin E levels in the liver [129], while alcoholics without cirrhosis generally have normal vitamin E levels. Oxidative stress plays a central role in alcohol-induced pathogenesis. Vitamin E supplementation restores the normal oxidative reduction status, reduces apoptosis, and prevents oxidative stress [130]. Vitamin E administration ameliorates learning and memory deficits induced by perinatal ethanol exposure and increases hippocampal brain-derived neurotrophic factor levels in a dose-dependent manner [131]. However, its usefulness in alcoholic liver disease remains unclear.

#### 4.2.5. Vitamin B1 (Thiamine)

Thiamine deficiency is less directly related to ALD; however, it is often a problem during early hospitalization. Thiamine deficiency is common in patients with alcohol consumption. Thiamine deficiency influences the cardiovascular, nervous, and immune systems, as observed in wet beriberi, dry beriberi, and Wernicke–Korsakoff syndrome [98]. Cognitive impairment may be an early consequence of a thiamine deficiency. Wernicke’s encephalopathy is a medical emergency that, if left untreated, can lead to permanent neurological deficits owing to its associated biochemical changes. Patients require emergency intramuscular or intravenous thiamine administration to prevent further progression, which often occurs with inadequate treatment [132,133]. Thiamine has a half-life of 96 min and should ideally be administered two to three times daily. For alcoholics at risk of Wernicke’s encephalopathy, 500 mg of thiamine hydrochloride diluted in 100 mL of saline is administered intravenously over 30 min. This is repeated three times daily for 2–3 days and is discontinued if the effect is inadequate. Patients on thiamine therapy should be given electrolyte supplementation, particularly magnesium and potassium because electrolytes are essential cofactors for proper enzyme function. Given the frequency of deficiency, vitamin B supplementation is likely to be beneficial. As vitamin B is water soluble, long-term supplementation, even at high doses, is considered safe [134].

#### 4.2.6. Folate

Serum folate levels are significantly lower in chronic alcoholics than in healthy individuals [135]. Alcohol consumption can decrease folate levels and cause folate deficiency when alcohol constitutes most of the calories consumed, as seen in malnourished alcoholics. Folate deficiency can cause macrocytic anemia [99] and a decrease in S-adenosylmethionine, leading to abnormal DNA methylation patterns and pathological changes in gene expression [100]. Aberrant DNA methylation is closely associated with the development of cancer. Low folate levels are associated with oxidative stress, liver injury, and cancer. Blood folate levels are inversely associated with liver injury and hepatocellular carcinoma development [101]. There is evidence that among alcohol drinkers, those with higher folic acid intake have lower risk of death from liver cancer than those with lower folic acid intake [102]. Increasing folic acid intake, especially in women who regularly consume alcohol, to levels above the current recommended dietary intake (>400 µg/day) may have important benefits in reducing the overall risk of chronic diseases [136].

#### 4.2.7. Zinc

Zinc (Zn) deficiency is common in all chronic liver diseases. Importantly, it causes many types of metabolic abnormalities, including insulin resistance, hepatic lipidosis, iron overload, and hepatic encephalopathy [106]. Approximately 90% of alcoholics have inadequate dietary zinc intake [137], and many patients with chronic alcoholism and AH have zinc deficiencies [138]. Given the high frequency of Zn deficiencies, Zn supplementation is likely to be beneficial. Zinc supplementation significantly improves the grades of hepatic encephalopathy and blood ammonia levels [106]. Low levels of Zn in the brain during alcohol intoxication may increase sensitivity to alcohol withdrawal-induced seizures, and Zn supplementation may prevent alcohol withdrawal symptoms [139]. Zinc has been shown to improve the intestinal mucosal barrier in animal models of ALD and small pilot clinical trials [140]. Oxidative stress is strongly associated with the pathogenesis of ALD, and there are reports showing that the combination of zinc acetate and rifaximin, a non-absorbable antibiotic, inhibits the development of fibrosis associated with ALD [141]. Additional studies are needed to determine whether preservation of intestinal barrier function with zinc administration is feasible in AH treatment. High doses of Zn can cause gastrointestinal symptoms. In addition, long-term high-dose Zn supplementation can lead to copper deficiencies. Zinc and copper levels should be routinely measured in patients with long-term zinc supplementation who develop anemia of unknown origin [69].

#### 4.2.8. Magnesium

Chronic alcohol abuse causes significant magnesium deficiencies [142]. Both total circulating magnesium and ionized circulating magnesium levels are markedly decreased in chronic alcohol use disorders. Skeletal muscle magnesium levels are also reduced [143]. Further, the normal response of the kidneys to hypomagnesemia is blunted [144,145]. Frequent assessments of blood magnesium levels are recommended for patients with chronic alcoholism. Magnesium supplementation would be beneficial for individuals with magnesium deficiency, such as the elderly and alcoholics [146]. Magnesium supplementation should be given along with thiamine supplementation, as mentioned above.

#### 4.2.9. Iron

Another contributing factor to the risk of ALD is high hepatic iron levels, which has been identified as a predictor of mortality in patients with alcoholic cirrhosis [112]. Iron overload is an independent factor in hepatocellular carcinoma progression [110]. High dietary iron intake may also increase the risk of hepatocellular carcinoma, suggesting that high iron intake may promote tumorigenesis [111]. Approximately half of ALD patients have hepatic iron overload, with elevated serum ferritin levels and early transferrin saturation. Low hepcidin levels, along with iron deposition in macrophages, are usually present in these patients [147]. As iron and alcohol independently cause oxidative stress, patients with hemochromatosis who consume alcohol exhibit cumulative liver damage, resulting in toxic damage to the liver due to alcohol-induced damage and increased intestinal iron absorption. This results in accelerated pathological progression to cirrhosis and increased predisposition to hepatocellular carcinoma [148]. Ferroptosis is an iron-dependent cell death pathway accompanied by massive lipid peroxidation, and increasing evidence shows that ferroptosis plays an important role in the pathogenesis of various types of liver diseases, including ALD [149]. In addition to phlebotomy and iron chelation, attempts are being made to improve fibrosis by regulating iron-related proteins; however, this is still in the research stage [112].

#### 4.2.10. Manganese

The role of manganese in ALD is not yet fully understood. However, manganese levels appear to be elevated in ALD. Manganese is considered to play an important role in the development of brain disorders related to alcohol abuse, such as hepatic encephalopathy [114]. Increased manganese deposition in the brain, particularly in the globus pallidus, has been observed in patients with hepatic encephalopathy [115]. In a study regarding trace element levels in cirrhotic patients, there was a significant increase in serum manganese levels in patients with Child–Pugh C cirrhosis compared to patients with Child–Pugh A and B cirrhosis [114]. This study cited biliary stasis as one of the causes of the elevated manganese levels. However, treatment recommendations for elevated manganese level are currently unknown.

#### 4.2.11. Copper

The relationship between copper levels and other metabolic conditions such as zinc is complex. Copper deficiency has been reported in several studies on nutritional deficiencies associated with ALD [68,150]. However, copper levels can also increase when zinc is deficient [114]. Copper deficiency in alcohol-dependent patients often correlates with central nervous system dysfunction and impairment. Copper deficiency also promotes dyslipidemia and increases oxidative stress. Whether copper supplementation is effective in ALD is inconclusive.

#### 4.2.12. Selenium

Selenium has antioxidant and anti-inflammatory functions. Its other functions include immune enhancement, reduction in cancer incidence, inhibition of tumor invasion and metastasis, and in the form of radiation and chemotherapy treatments in clinical applications [151]. Selenium levels were found to be low in alcoholics with ALD [152]. Additionally, low selenium may cause ballooning of hepatocytes [116]. Notably, selenium supplementation may be as effective as antioxidant therapy [153].

## 5. Conclusions

Nutritional strategies may be helpful for the treatment of alcoholics, including those with ALD. The most important treatment for ALD is abstinence from alcohol; however, alcohol is highly addictive, and thus, abstinence is often difficult. Patients with ALD require multidisciplinary team care. In particular, advanced ALD requires appropriate nutritional therapy according to the disease stage.

In AH patients, for example, a high-protein, high-energy diet should be administered enterally to AH patients without gastrointestinal bleeding complications; it is recommended that AH patients consume 1.2–1.5 g/kg protein and 35–40 Kcal/kg calories per day. Thiamine and other B vitamins should be administered during nutritional supplementation. Supplementation with trace elements, including zinc, should also be considered. When anemia is observed, supplementation with iron and folic acid should also be considered. Deficiencies in vitamins and trace elements commonly found in ALD often cause irreversible damage and should always be considered in ALD treatment. Supplementation is necessary when these deficiencies are suspected. On the other hand, ALD patients were often found to be undernourished in the past; however, now they are often over nourished, consequently requiring their nutritional therapies to be modified. This review is useful given the scarcity of studies that mention nutritional therapy for each phase of ALD or vitamin and trace element replacement therapy in the current scientific literature.

## Figures and Tables

**Table 1:** Recommended nutritional therapy for ALDs.

Alcoholic Liver Diseases	Recommended Total Energy and Nutrient Intake	Reference(s)
Alcoholic hepatitis	Total energy: 35–40 kcal/kg/day	[25]
	Protein: 1.2–1.5 g/kg/day	
Alcoholic fatty liver or steatosis	Total energy: 20–40 kcal/kg/day	[25,26]
	Protein: 1.0–1.5 g/kg/day	
	Carbohydrate: 50–60% of total energy	
	Lipid (low saturated fatty acid): 15–20% of total energy	
Alcoholic cirrhosis	Total energy: 25–40 kcal/kg/day including LES	[25,27,28]
	Total energy: 25 kcal/kg/day in the presence of glucose intolerance	
	Protein: 1.2–1.5 g/kg/day including BCAA preparations	

## Data Availability

Not applicable.

## Abbreviations

ADH	alcohol dehydrogenase
AFL	alcoholic fatty liver
AH	alcoholic hepatitis
ALD	alcoholic liver disease
ALDH	aldehyde dehydrogenase
BCAA	branched-chain amino acid
BMI	body mass index
BTR	BCAA-to-tyrosine ratio
CYP2E1	cytochrome P450 2E1
FFA	free fatty acids
Ile	isoleucine
Leu	leucine
MAFLD	metabolic dysfunction-associated fatty liver disease
NASH	nonalcoholic steatohepatitis
NOX	NADPH oxidase
PEM	protein–energy malnutrition
REE	resting energy expenditure
ROS	reactive oxygen species
TG	triglycerides
TLR4	Toll-like receptor 4
Val	valine
Zn	zinc
